# Estimating and Analyzing Savannah Phenology with a Lagged Time Series Model

**DOI:** 10.1371/journal.pone.0154615

**Published:** 2016-04-29

**Authors:** Niklas Boke-Olén, Veiko Lehsten, Jonas Ardö, Jason Beringer, Lars Eklundh, Thomas Holst, Elmar Veenendaal, Torbern Tagesson

**Affiliations:** 1 Department of Physical Geography and Ecosystem Science, Lund University, Lund, Sweden; 2 School of Earth and Environment (SEE), The University of Western Australia, Crawley, Australia; 3 Centre for Ecosystem Studies, University of Wageningen, Wageningen, the Netherlands; 4 Department of Geosciences and Natural Resource Management, University of Copenhagen, Copenhagen, Denmark; The University of Melbourne, AUSTRALIA

## Abstract

Savannah regions are predicted to undergo changes in precipitation patterns according to current climate change projections. This change will affect leaf phenology, which controls net primary productivity. It is of importance to study this since savannahs play an important role in the global carbon cycle due to their areal coverage and can have an effect on the food security in regions that depend on subsistence farming. In this study we investigate how soil moisture, mean annual precipitation, and day length control savannah phenology by developing a lagged time series model. The model uses climate data for 15 flux tower sites across four continents, and normalized difference vegetation index from satellite to optimize a statistical phenological model. We show that all three variables can be used to estimate savannah phenology on a global scale. However, it was not possible to create a simplified savannah model that works equally well for all sites on the global scale without inclusion of more site specific parameters. The simplified model showed no bias towards tree cover or between continents and resulted in a cross-validated r^2^ of 0.6 and root mean squared error of 0.1. We therefore expect similar average results when applying the model to other savannah areas and further expect that it could be used to estimate the productivity of savannah regions.

## Introduction

Leaf phenology of savannah ecosystems is an important driver of the carbon cycle by affecting the timing and amount of the primary production at both regional and global scales [1]. Leaf phenology is a term used to describe natural events such as budburst and leaf fall, and here we use it to describe the full seasonal cycle of changing leaf states. While the environmental controls on leaf phenology have been shown to vary with geographical region [2], the focus of most studies has been on temperate ecosystems [3] where temperature and photoperiod duration are the main controlling variables [4,5]. Few phenological studies have been undertaken in water-limited ecosystems, such as savannahs, even though they comprise around half of the world’s terrestrial ecosystems in terms of area [6].

Savannahs are particularly important because they are populated with societies dependent mainly on subsistence farming in some parts of the world. This is mainly true for African savannahs, where harvest loss due to unsuitable weather conditions during (mostly short) wet seasons can have major effects on food security and economic growth [7]. Apart from their importance for food production, savannahs play an important role in the global carbon cycle due to their total areal coverage [8]. However, savannah ecosystem function and phenology are currently poorly characterized in global vegetation models [9]. For example, in the dynamic vegetation model LPJ-GUESS [10], savannah trees and grasses are considered to be in full leaf cover if the ratio between water demand and water supply is above a fixed threshold. As the vegetation in the model never experiences water stress when in full leaf, this implementation potentially leads to an overestimation of photosynthesis. This simplified approach also causes trees and grasses to be in full leaf cover immediately after any sufficiently strong rain event. This method of representing savannah leaf phenology is similar to other dynamic vegetation models [11,12] and indicates a need for an improved savannah phenology representation. Previously, savannah phenology models mainly focused on predicting the onset of the growing season [13–15] and phenological changes related to rainfall variability [16]. However, Jolly et al. [2] developed a global phenology model using vapour pressure deficit, temperature, and day length. Their model produced a growing season index (GSI) that had a high overall correlation with the normalized difference vegetation index (NDVI), however when combined across multiple sites the model exhibited low correlations between absolute values of NDVI and GSI, which limits its applicability to other savannah ecosystems in different regions.

In this study, we use satellite-derived NDVI to represent the seasonal cycle of leaf phenology. NDVI is a spectral index of vegetation greenness [17], and is commonly used for observing vegetation seasonality for regions where field data are sparse [18,19]. Advantages of using remotely sensed NDVI is that it generates quasi-continuous year-round estimates of vegetation greenness. However, the NDVI signal does not separate trees and grasses. This might pose a challenge because trees and grass can have different phenological cycles [15], which vary dependent on species and location. For example, the onset of grass growth has been shown to be related to water availability, soil moisture, and day length [13,20,21], whereas tree phenology is influenced mostly by temperature and day length [13,22,23]. This difference in grass and tree phenology is likely the result of different strategies of when to start developing leaves [13] and how to use water reservoirs (shallow vs. deep root systems). Furthermore, savannah tree vegetation can display different survival strategies and leaf habits between continents. For example, evergreen savannahs in Australia might occur due to different leaf traits compared to other continents [24]. This shows the knowledge gap in savannah phenology studies regarding the underlying climatic mechanisms [3] and also indicates that vegetation has developed to use different strategies in different places which can limit a global model that has not been parameterized to take those specific strategies into account.

The objective of this study is to develop a global phenological model explaining climatic controls on savannah vegetation. This is essential since vegetation generates feedbacks to the climate system and affects the water cycle, surface albedo, energy fluxes and surface roughness [3,25]. Thus, improving our ability to model savannah phenology is crucial for evaluating how projected climate change will influence the length of the growing season, its timing, and productivity [26]. Here, we present a global phenological model linking in-situ climate data to leaf phenology for 15 sites located on four continents. This approach allows the model to be incorporated into global vegetation models or used as a prognostic tool to estimate ecosystem greenness for savannah sites.

## Methods

### Data

**Fig 1:**
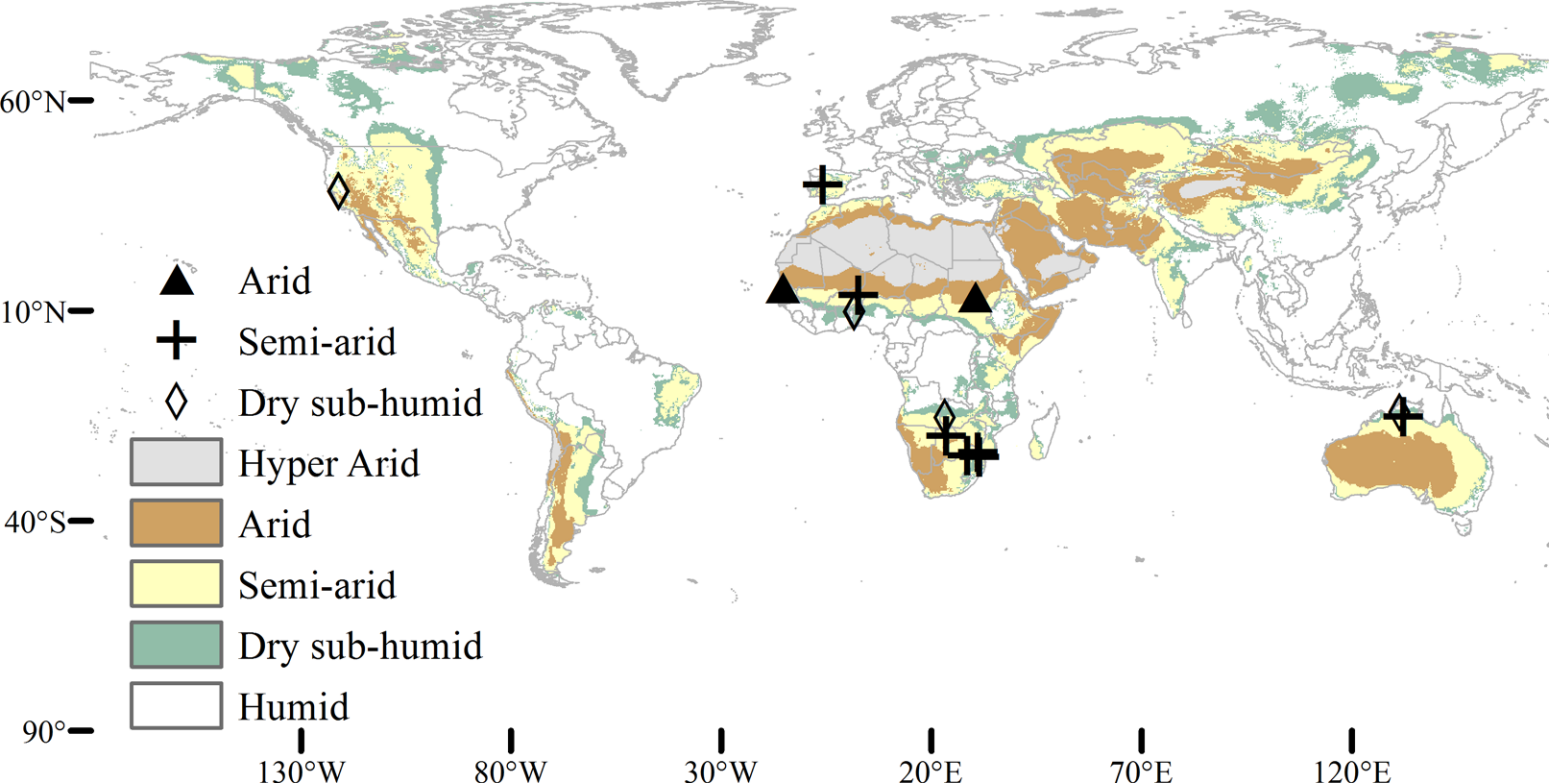
Map showing site locations of study. Sites used for study are located in Africa (10), Australia (2), North America (2), and Europe (1).

We used soil moisture and temperature data for 15 sites located in Africa (10), Australia (2), Europe (1) and North America (2) (Fig 1, Table 1). The sites were selected using an aridity index (AI) [27] and we chose for each site the time period with the most complete record (Table 1). AI is defined as the ratio between average potential evapotranspiration and mean annual precipitation for the period 1950–2000. Only sites with both tree and grass and classified as arid (0.03<AI<0.2), semi-arid (0.2< = AI<0.5), or dry sub-humid (0.5< = AI<0.65) were chosen.

**Table 1:** Site information.

Name	Country	Lat	Lon	Tree Cover (%)	Aridity index	Soil moisture importance (%)^a^	Reference
Bira	Benin	9.82	1.72	32	0.62	32	[28]
Dahra	Senegal	15.4	-15.43	6	0.19	57	[29]
Daly	Australia	-14.16	131.39	96	0.58	35	[30]
Demokeya	Sudan	13.28	30.48	12	0.14	62	[31]
Dry River	Australia	-15.26	132.37	44	0.41	45	[32]
Las Majadas del Tietar	Spain	39.94	-5.77	36	0.38	52	[33]
Malopeni	South Africa	-23.83	31.21	40	0.31	36	[34]
Maun	Botswana	-19.92	23.59	72	0.24	36	[35]
Mongu	Zambia	-15.44	23.25	53	0.5	38	[36]
Nalohou	Benin	9.75	1.61	8	0.62	42	[28]
Nylsvley	South Africa	-24.65	28.7	50	0.38	24	[37]
Skukuza	South Africa	-25.02	31.5	16	0.43	39	[38,39]
Tonzi Ranch	USA	38.43	-121	42	0.53	46	[40]
Vaira Ranch	USA	38.41	-121	54	0.53	44	[40]
Wankama Fallow	Niger	13.65	2.63	12	0.21	54	[41,42]

^a^Soil moisture importance in modelling phenology as calculated in section 2.3. Importance separated between day length and soil moisture, day length importance = 100 –soil moisture importance.

The climate data utilized in this study were in-situ volumetric soil moisture in the layer closest to the surface (usually 5–10 cm) and air temperature. In-situ air temperature was used for a majority of the site except for Wankama, Bira, Nalohou, and Nylvsley where a global meteorological dataset was used to derive the air temperature (Water and Global Change (WATCH) Forcing Data methodology applied to ERA-Interim data (WFDEI) [43]). The long term mean annual precipitation (MAP) was obtained from site descriptions available or calculated from the climate data. Day length was calculated using site latitude and date. We included day length, minimum temperature and soil moisture since it has previously been shown to influence the leaf phenology of savannahs [13,23]. However, since day length and minimum temperature are known to be correlated we removed minimum temperature as it showed on average the lowest correlation (r = 0.13) with NDVI.

NDVI was obtained from two MODerate Imaging Spectroradiometer (MODIS) vegetation index products, MOD13Q1 and MYD13Q1 [44]. Both NDVI products come as 16 day composite data sets. The composite means that the NDVI value for each pixel was recorded on one day within those 16 days. We used the real acquisition date for the NDVI products, which can vary between nearby pixels dependent on for example cloud cover. Therefore, to avoid averaging over different acquisition dates, we used a single pixel of NDVI (size 250 m x 250 m) covering the location for each of the sites. We gap-filled the NDVI data with a linear interpolation and resampled it to a temporal resolution of 8-days. The NDVI data were finally smoothed to remove disturbances using a Savitzky-Golay smoothing filter in a similar approach as Jönsson and Eklundh [45]. In the filtering, the midpoint values were updated using a second degree polynomial fit applied to a seven time step moving window. Filtered NDVI data were then checked for large changes to avoid removing valid rapid changes that can be typical for savannahs. If a large change (>0.08 dimensionless NDVI unit) was detected between a data point and its neighbors in time, that point was instead filtered with a 3-timestep moving window. An 8-day median soil moisture and maximum day length were calculated from the daily climatic data and matched to the time steps of the NDVI.

Tree canopy cover was estimated using Google Earth imagery by visually inspecting 50 randomly selected points within the 250x250 m NDVI pixel for presence of tree cover. An online tool (i-Tree Canopy v 6.1,[46]) was used to sample the points, display the Google Earth imagery available during January-February 2015, and identify tree cover. The exact date of the aerial imagery was not available but assumed to not affect the result since the tree crowns were visible independent on its leaf cover. Google Earth was used since other tested remote sensing products of tree cover gave some irregularities in the result when compared with site descriptions. For example MOD44B tree cover [47] indicated a tree cover even at two pure grassland sites (not used in this study) and was therefore deemed as unreliable for the purpose of our study.

### Phenology models

A statistical phenology model was developed and evaluated. Since previous studies have shown a significant lag between climatic events and vegetation activity [48,49] we used an explanatory approach which allowed the most important variables and their time lag to be selected as model parameters. The time lags were introduced to account for a potential mismatch between the climatic variables and leaf development (photosynthetic activity).

**Fig 2:**
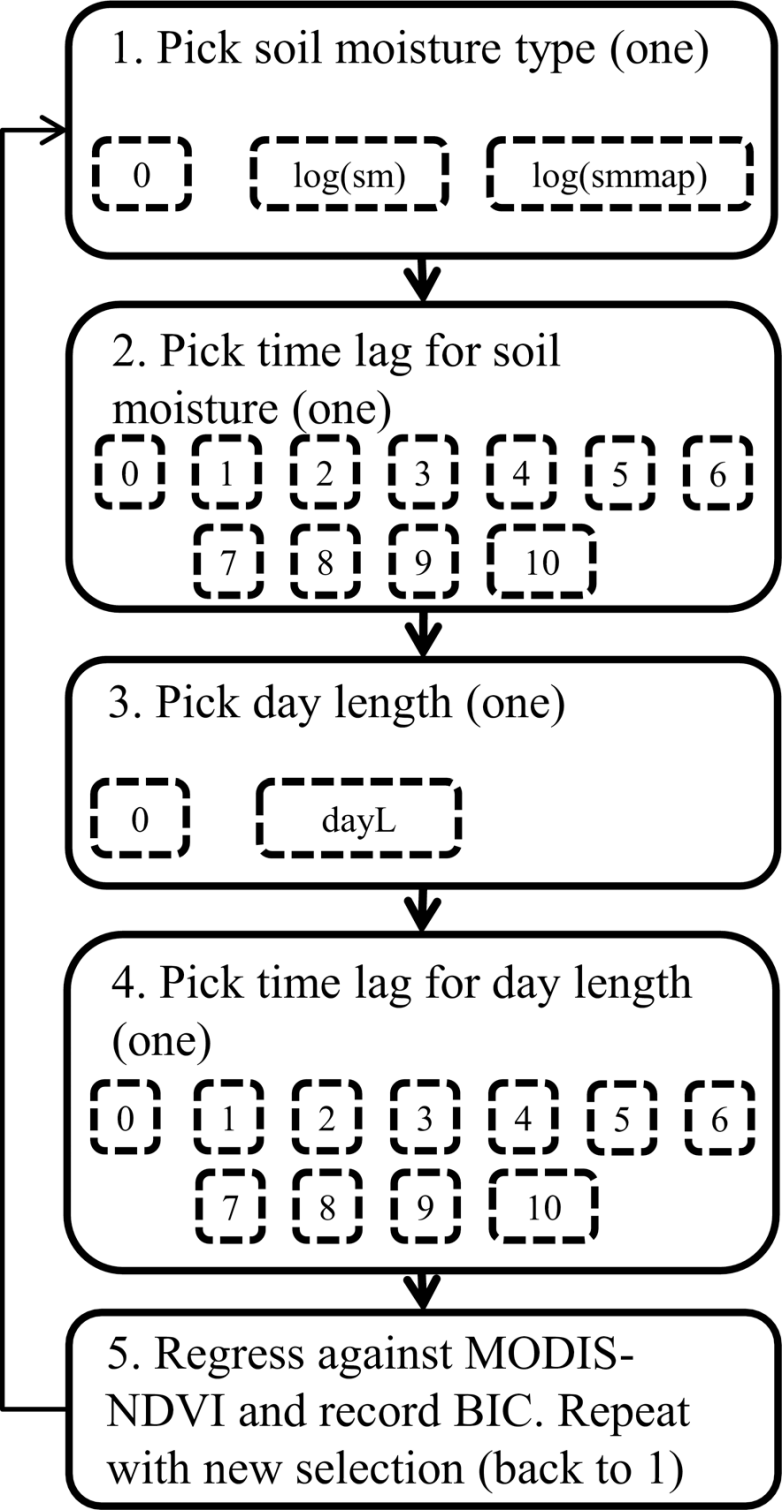
Visual representation of the steps taken in model selection. The five steps shown are repeated until all combinations are found. DayL is day length in hours, log(sm) is the natural logarithm of soil moisture, and smmap is soil moisture multiplied with mean annual precipitation (m/year). BIC is the Bayesian information Criterion used to evaluate.

The developed model was a lagged multiple variable time series regression model. The selection of the best regression model was done by assessing all possible combinations of the two variables and their time lags by repeating the model selection steps shown in Fig 2. The models were restricted to include each variable (soil moisture and day length) only once, and the maximum considered time lag was 10 time steps (80 days). Soil moisture was natural log transformed and allowed to be multiplied with mean annual precipitation (MAP). The alterations were done since MAP has been shown to be linearly related to NDVI [50] and an initial analysis of the data showed an exponential behavior. The best model was assumed to be the one with lowest Bayesian Information Criterion (BIC) [51]. The BIC was chosen as a model selection tool since it is known to show whether additional model parameters result in a better model or are simply over-fitting the model [51].

### Variable importance

To analyze site-related differences and to assess model performance, the variable importance of soil moisture and day length was estimated for each site using the *average over ordering of regressors* function included in the R package *relaimpo* [52]. The function provides information on how much each variable contributes to the total coefficient of determination (r^2^) of the linear regression model while also accounting for differences in the ordering. Soil moisture and day length were each included in the linear regression as a combination of all lags of up to 10 time steps (80 days). Finally, the contribution of each variable and its lags to the r^2^ were summed to get a single measure of the importance of the variable.

### Model evaluation

The model was tested for its sensitivity to adding more sites by estimating the root mean squared error (RMSE) 1000 times for each model size (i.e. number of sites included in the model parameterization), selecting the sites and their order randomly. This test was done to ensure that the selected model was stable with respect to the incorporated sites and did not change substantially when adding more sites compared to a parameter estimation based on fewer sites. The model was also evaluated using a cross-validation hold-out method which is a way of dividing the data into training and evaluation subsets [53]. In this case 12 of the sites and their order were used randomly as training and the remaining three sites as evaluation data, and this process was repeated 1000 times. The evaluation subset was used to calculate the RMSE, r^2^, and variance inflation factor (VIF, to assess multicollinearity) in each repetition, and the average value of those parameters were assumed to represent the performance of the model. The result is also the expected model performance when the model is used for other savannah sites or regions not being represented in the model development. The capability of the model to estimate the start of season (SOS) was compared to SOS estimated from MODIS-NDVI. SOS was defined as the time step closest to the midpoint between maximum and preceding minimum NDVI value for each growing cycle. The modelled NDVI was filtered in the same way as MODIS-NDVI with the Savitzky-Golay filtering method adapted from Jönsson and Eklundh [45]. Finally, the amplitude of the model was compared with the amplitude of MODIS-NDVI (per site). The amplitude was calculated by subtracting the minimum NDVI value from the maximum NDVI value.

## Results

### Phenology model

The development of the phenological regression model showed that day length (dayL, in hours), lagged with two time steps (16 days, indicated with subscript 2), and natural log transformed soil moisture (sm, in volumetric water content %), lagged with two time steps (16 days, indicated with subscript 2), multiplied with mean annual precipitation (MAP, in m/year) were the most important variables for the model (Eq 1).

$$NDVI=0.12\cdot log({sm}_{2}\cdot MAP)+0.01\cdot {dayL}_{2}+0.22$$ (1)

### Variable importance

**Fig 3:**
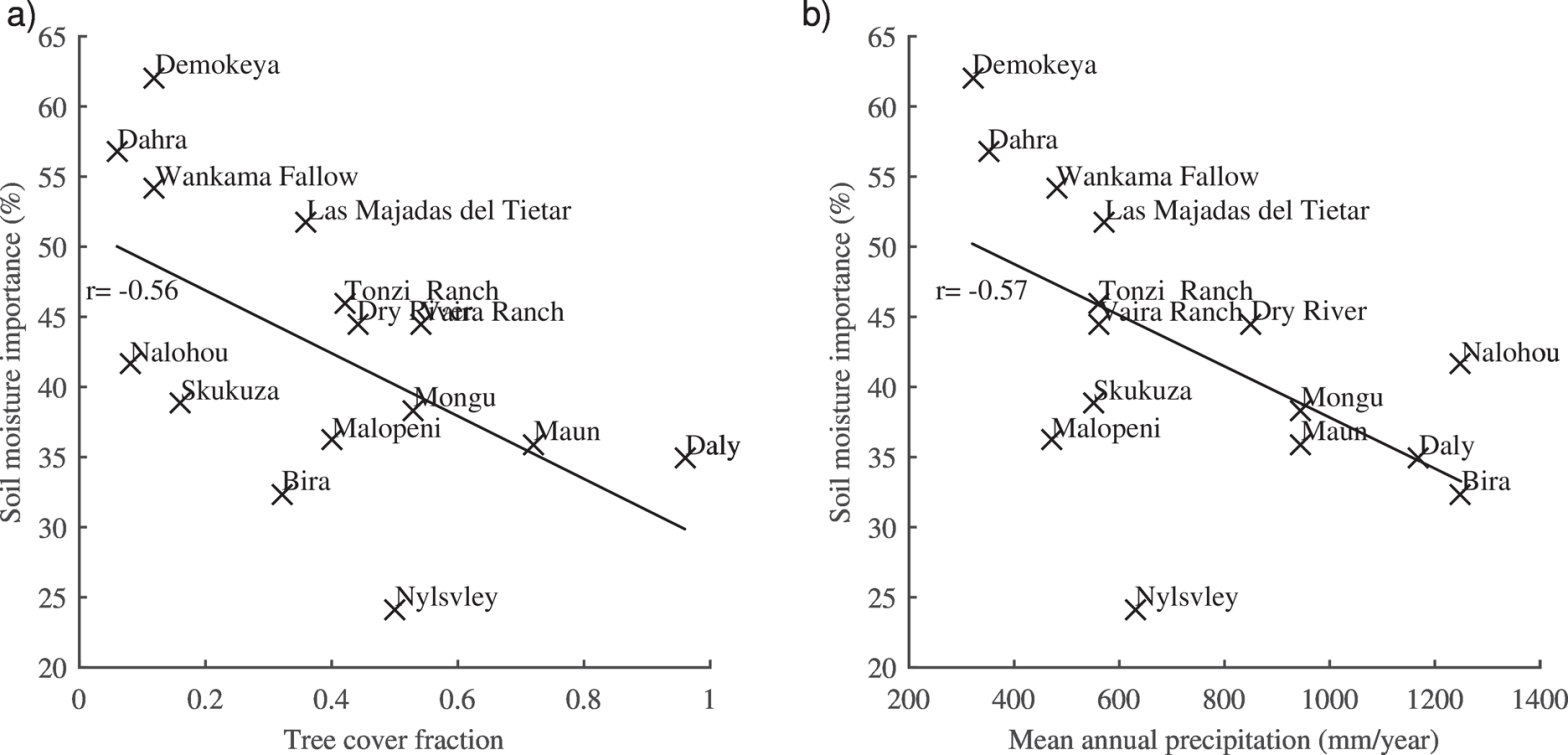
Variable importance analysis. a) Soil moisture variable importance related to tree cover. b) Soil moisture variable importance related to mean annual precipitation. Correlation coefficients (r) are for both panels shown next to the linear regression line. Day length importance (in %) can be calculated by taking 100-(soil moisture importance).

The average importance of soil moisture for all sites was 42.8% and it showed a strong negative correlation (r = -0.56, p = 0.03) to tree cover (Fig 3A) and a strong negative correlation (r = -0.57, p = 0.03) to mean annual precipitation (MAP) (Fig 3B).

### Model evaluation

**Fig 4:**
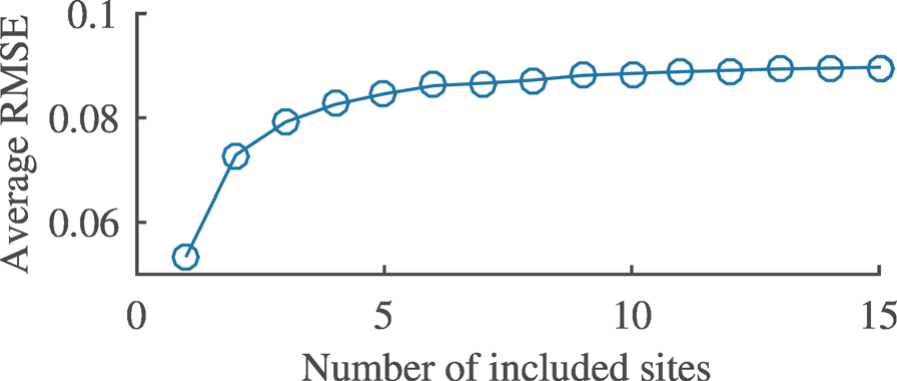
Influence of the number of included sites in the model development. Average root mean squared error (RMSE) of the model with respect to the number of included sites. Statistics are based on developing the model 1000 times for each included sites size.

The model (Eq 1) was evaluated by testing its sensitivity to the number of sites included in the model, i.e. the size of the data pool used to develop the model. When the model parameterisation process included more sites the RMSE increased but reached saturation when around seven or more sites were included (Fig 4).

**Fig 5:**
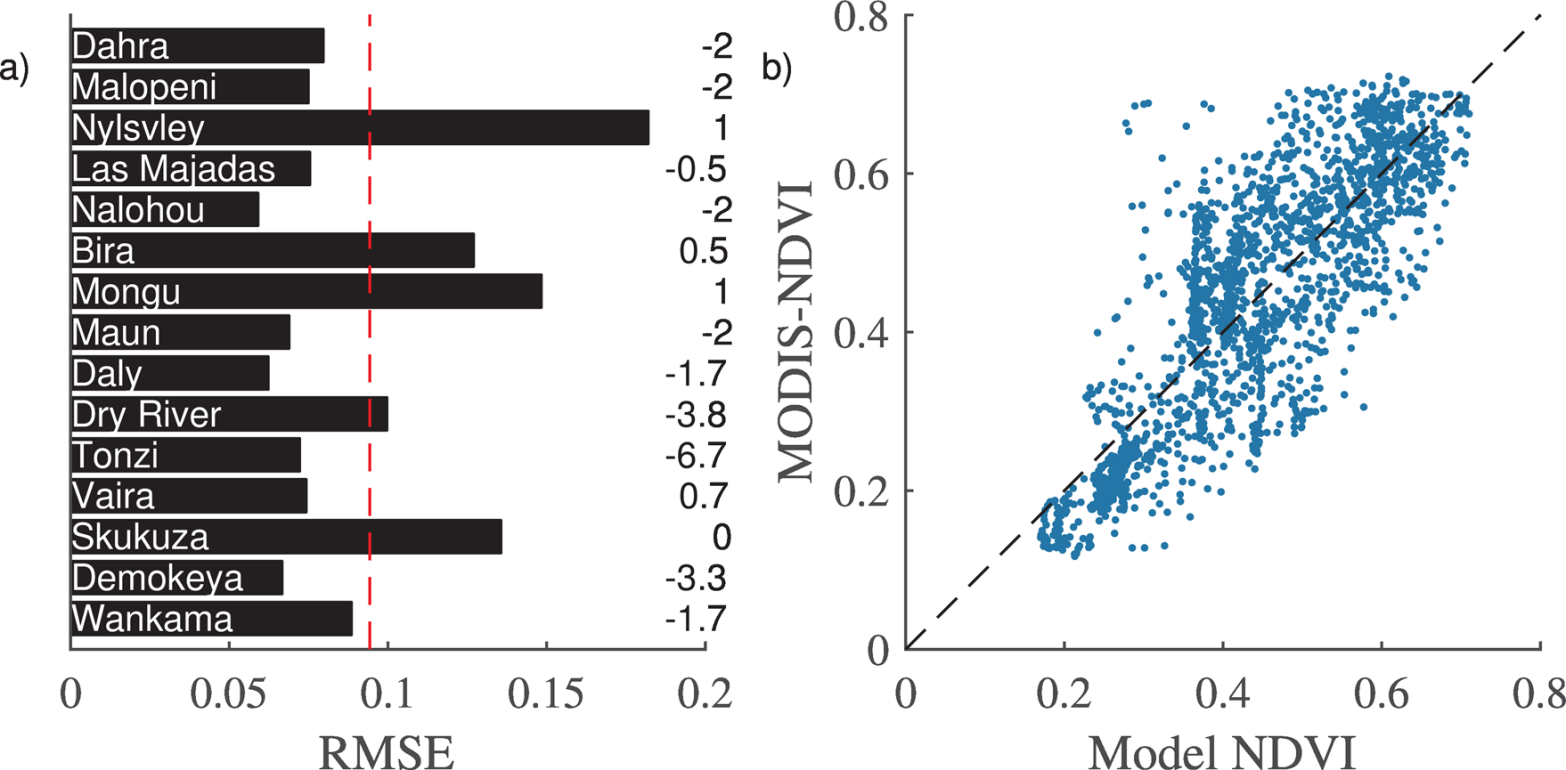
Model evaluation. a) Model evaluation at each site showing the root mean squared error (RMSE) to MODIS-NDVI. The dashed line shows the average value for all sites. Numbers to the right indicate SOS differences between model NDVI and MODIS-NDVI in 8 day time steps (SOS global model–SOS MODIS). b) MODIS-NDVI vs Model NDVI for all sites. Dashed line represents the 1:1 line. SOS: start of season.

**Fig 6:**
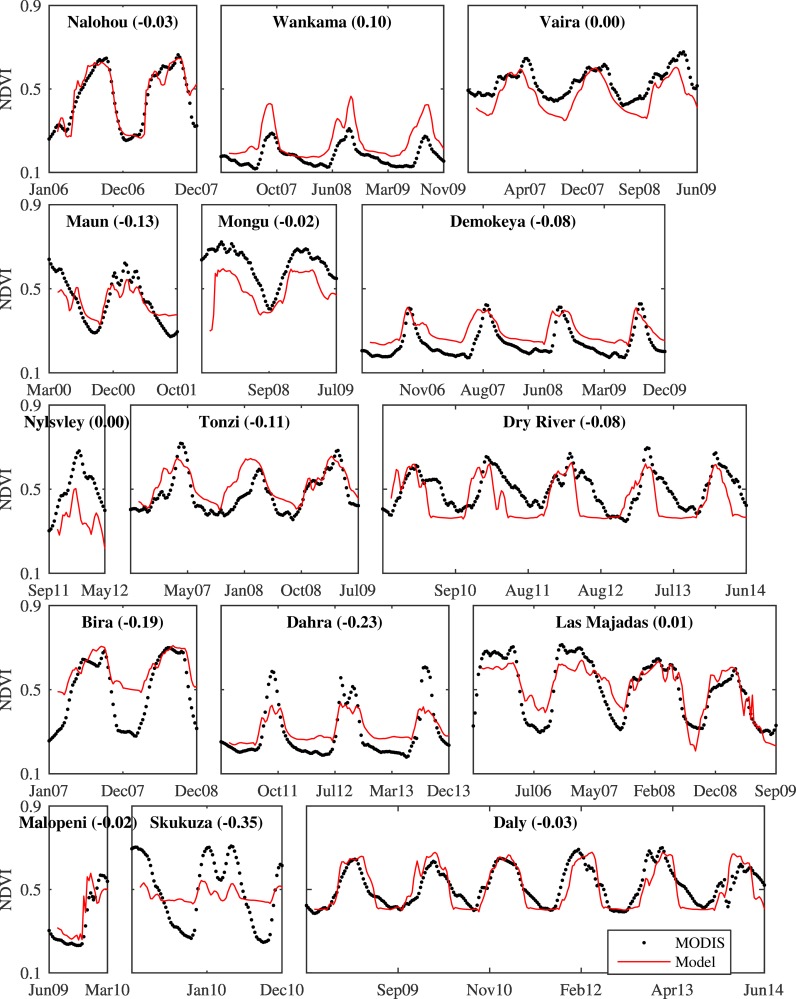
Time series of model (red line) and MODIS-NDVI (dots). Data has been filtered with a modified Savitsky-Golay filter as described in the methods section. Site name and global model amplitude error (model amplitude—MODIS amplitude) is shown as title for each subplot.

The cross validation with MODIS-NDVI showed an average RMSE of 0.10 ± 0.02,an average coefficient of determination (r^2^) of 0.60 ± 0.18, and an average VIF of 1.20 ± 0.20 when evaluated randomly 1000 times. The VIF result indicates that the multicollinearity between the two explanatory variables day length at time lag 2 and soil moisture at time lag 2 (Eq 1) is very low. The model produced some inconsistent results for Nylvsley, Mongu, Skukuza, and Bira with a RMSE above 0.12 (Fig 5A). The RMSE showed a strong negative correlation (r = -0.54) to soil moisture importance and no correlation with tree cover (r = 0.004) or MAP (r = 0.05). The model gave a start of season (SOS) that was on average 1.5 time steps (12 days) earlier than the SOS from MODIS-NDVI (Fig 5A) and the majority (12) of the sites had a SOS error lower or equal to ±2 time steps (16 days). On average the amplitude of NDVI was 0.077 (dimensionless NDVI unit) lower for the model compared with the MODIS-NDVI amplitude (Fig 6).

The model showed more variation in the upper half of the NDVI values and an over estimation in the lower part (Fig 5B). For some of the sites it provided an acceptable temporal agreement with MODIS-NDVI (Fig 6). But for some of the sites (Skukuza, Wankama, and Dahra) the amplitude error was above 50% of MODIS-NDVI amplitude (Fig 6) showing that the model cannot capture the MODIS-NDVI time series equally well for all of the sites. However, when evaluating the model performance based on RMSE, SOS error, and amplitude error five of the sites (Las Majadas, Daly, Malopeni, Nalohou, and Vaira Ranch) outperforms the others with RMSE below 0.1, SOS error less or equal to ±2 time steps (16 days) and amplitude error ±0.1 (dimensionless NDVI units). Those five sites are representing all of the 4 continents and have a tree cover ranging from 8% to 96% with an average value of 46.8%.

## Discussion

Our study demonstrates the difficulties of creating a common simplified model to estimate savannah phenology for 15 sites on four continents. However, the simplified approach makes the presented model easy to use for new applications. The RMSE did not increase when increasing the number of sites above seven in the model development. Therefore, we expect the cross-validated result (RMSE = 0.10 ± 0.02, r^2^ = 0.60 ± 0.18) to be a representative average range for the model when applying it to new savannah sites or areas. The presented model only requires day length, soil moisture and MAP. Based on our results, we estimate that MAP affects the absolute magnitude of the NDVI, soil moisture sets the temporal dynamics, and day length accounts for some of the site specific differences related to the phenological strategies of trees and grasses.

We showed that the soil moisture importance was related to tree cover with a moderate negative correlation (r = -0.56). However, since MAP is known to have an influence on tree cover for savannahs [54] we expect this to be caused by the correlation between soil moisture importance and MAP (r = -0.57). Thus for drier sites the amount of water and timing is more important compared to sites that have more plant available water or higher tree cover with a deeper root system. Since we found no correlation between RMSE and mean annual precipitation (r = 0.05) or tree cover (r = 0.004) we expect that some of the site specific differences regarding this are captured by the inclusion of mean annual precipitation as a model parameter. In Contrast we found that RMSE was strongly negatively correlated (r = -0.54) to soil moisture importance indicating a positive bias (less RMSE) in the result towards the sites where soil moisture was more important. This result was mainly influenced by four of the sites having a RMSE above 0.12 (Nylvsley, Mongu, Skukuza, and Bira) in combination with a low soil moisture importance. We attribute this result again to site differences which our simplified approach has not included in the model development. Despite this those four sites all showed a lower than average SOS error that indicates that the temporal signal was correctly captured but the error in amplitude or bias in the average value was mainly causing the high RMSE. The error in amplitude and bias in average are hypothesized to be partly related to differences in soil types. Soil types have previously been shown to have an effect on the relation between soil moisture and NDVI, via the so called moisture use efficiency [55] which supports our hypothesis. Soil type was not included in this study since that would increase the complexity of the model.

The analysis of the SOS error showed that the model on average managed reasonably well to estimate SOS (-12 days). However, three of the sites (Demokeya, Dry River, and Tonzi), showed a much larger SOS error compared to the remaining sites. We attribute the larger SOS error for those sites to the variability originating from different site specific parameters not considered within this study. This could for example be due to differences in species composition, soil depth, ground water access, grazing, human activity, or fire. We assume that fire and human activities are similar for all sites during the measurements used in this study. Un-managed fire is most likely prevented due to the management of the measurement sites, and human activity is restricted. However, differences in species composition and grazing have not been considered in this study. As grazing has even been shown to affect NDVI differently for different sites [56] this would have an influence on the assumptions made in model development and would most likely increase the uncertainty of the model results. We also hypothesize that the overestimation of NDVI values in the lower half of the parameter space (Fig 5B) could be caused by grazing. However, since we have only partial information on grazing or species composition we were not able to test this. Furthermore, inclusion of grazing information would limit the model applicability since this information would not be available at a global scale. Even though it has been shown that savannahs on different continents function differently in response to rainfall and fire [57] we do not find any bias in our model result towards any continent indicating that the inclusion of mean annual precipitation as a model parameter might capture some of the continental variability.

Previous works on savannah phenological models have mainly focused on the regional scale. For example, Choler et al. [58] created a phenology model for Australian semi-arid grasslands using soil moisture. Their resulting model had an average r^2^ of 0.73 which is higher than the cross-validated result of the global model (r^2^ = 0.60) developed in this study but is created at a regional scale which cannot be directly transferred to the global scale, and thus lacks a global applicability. It is also important to note that remotely sensed NDVI itself is a proxy for the vegetation greenness on the ground and does not provide any species specific information that can be used to differentiate between tree and grass. Pooling these two growth forms together in the phenology signal might have resulted in obscuring some of the site differences. By relating our model result to tree cover we tested for some of these differences. To improve the global model a separation of trees and grasses should be considered, which would increase the model complexity but has also been shown to require a very good parameterization in order to increase model performance [59]. Furthermore, at a global scale there is no reliable dataset available to perform such a analysis. Therefore we decided to develop a model without such a separation.

The variables used in our model development have been considered to be equally important over the season, which might have been an oversimplification. However, when we examined the possibility of developing separate models for the wet and dry season the results only improved marginally (2%–3%, data not shown) compared to using the presented model which is only including half of the parameters. To improve on this, an even more complex model for identifying different parts of the season would be required. However, in this study we showed that even with a simple model using only two variables and their lags we can already explain a large part of the variability. If we developed a more complex model, more parameters would be required and the systematic error would most likely increase. Furthermore, the aim in this study was to generate a simple model that can easily be applied to other studies which further prevents the inclusion of more complexity.

The presented work highlights the difficulties of developing a global savannah phenological model. Despite the deviation from remotely sensed data we assume that if the presented model were integrated into a dynamic vegetation model this would improve global carbon estimates given the overly simplistic way that savannah phenology currently is handled in many DGVMs. Given the close link between NDVI and leaf area index as well as albedo (at least within savannahs which have a relatively low leaf area index) the presented model can also serve as a sub-model in simulations of regional climate models, which often still use static albedo [60]. The main benefit of taking the presented approach to model savannah vegetation is that it covers the whole growing season and is directly comparable to NDVI. NDVI is used in a number of studies as a proxy for photosynthetic light uptake, leaf area index [61,62], gross primary productivity [63], and seasonal characteristics such as start of growing season, growing season duration, and end of season for which highly developed algorithms exist, e.g. TIMESAT [45].

## Conclusion

This study addressed the climate-vegetation interaction of savannahs by using day length and soil moisture to model leaf phenology. We used NDVI as a proxy of leaf phenology and showed that the time-series of NDVI was best captured using a lagged log transformation of soil moisture and MAP together with day length. We showed that it was not possible to generate a simplified savannah model that gave perfect results for all sites with a small set of parameters that also has a good global availability. However, we show that it is possible to get a sufficient average result. The presented model can potentially be integrated into a dynamic vegetation model or used to generate rough estimates of NDVI from measured or predicted soil moisture and MAP data to predict savannah photosynthetic light uptake, gross primary productivity, albedo, and seasonal characteristics.
